# *Salmonella typhimurium* necrotizing fasciitis: a case report

**DOI:** 10.1186/s13256-023-04090-x

**Published:** 2023-08-13

**Authors:** Radwan El Othman, Fatima Allaw, Antoine Kharsa, Souheil Hallit

**Affiliations:** 1 Department of Internal Medicine, American University of Beirut Medical Center (AUBMC), Beirut, Lebanon; 2 Division of Infectious Diseases, American University of Beirut Medical Center (AUBMC), Beirut, Lebanon; 3 School of Medicine and Medical Sciences, Holy Spirit University of Kaslik, P.O. Box 446, Jounieh, Lebanon; 4 Applied Science Research Center, Applied Science Private University, Amman, Jordan; 5 Research Department, Psychiatric Hospital of the Cross, Jal Eddib, Lebanon

**Keywords:** *Salmonella*, Salmonellosis, *Salmonella typhimurium*, *Salmonella* non-typhi, Bacteremia, Necrotizing fasciitis, *Fusarium*, Immunodeficiency, Case report

## Abstract

**Background:**

Necrotizing fasciitis is an aggressive disease that causes necrosis in the muscular fascia and subcutaneous tissues. The infection spreads rapidly along the fascia and perifascial planes, followed by extension of the infection to nearby soft tissues and muscles. Necrotizing fasciitis can be attributed to different pathogens, namely *Staphylococcus aureus*, group A streptococci, and *Clostridium perfringes*. Only a few cases of skin and soft tissue infections from *Salmonella* species have been reported to date. Herein we report a case of *Salmonella* non-typhi necrotizing fasciitis, an exceedingly rare entity. This case report may serve as a potential management plan in similar cases in light of the scarcity of evidence.

**Case presentation:**

A 20-year-old Caucasian male patient with congenital cardiac anomaly presented with diarrhea and unilateral lower extremity cellulitis causing septic shock. Cultures from blood and the bullae associated with the lower extremity cellulitis grew *Salmonella typhimurium*. Surgical debridement was performed. Intraoperative tissue cultures were positive for *Salmonella typhimurium*, and surgical pathology confirmed the diagnosis of necrotizing fasciitis. After a total of 6 weeks of appropriate antimicrobial therapy, another surgical debridement was executed for poor wound healing. New intraoperative cultures grew *Fusarium* species, and the patient received voriconazole with an adequate response. Immunologic studies showed humoral and cellular immunodeficiency.

**Conclusion:**

It is important to maintain a high index of suspicion for rare entities that can cause skin and soft tissue infections, such as *Salmonella* non-typhi, in particular in immunosuppressed patients where a delay in diagnosis and management may have significant morbidity and mortality.

## Background

Necrotizing fasciitis (NF) is an aggressive skin and soft tissue infection (SSTI) that causes necrosis in muscular fascia and subcutaneous tissues. Infection travels along the fascial plane, which has a low blood supply. Hence, the overlying tissues are initially spared, while the fascia and preifascial planes are necrotizing, which might delay diagnosis. That is followed by an extension of infection to nearby soft tissues and muscles [1]. NF is a life-threatening condition that requires prompt surgical intervention [2]. Causative microbiologic pathogens are either polymicrobial or monomicrobial. Most cases are attributed to polymicrobial involvement and usually occur in immunocompromised patients, while monomicrobial cases are rare, seen mostly after a penetrating trauma injury. The usual pathologic isolates are *Staphylococcus aureus*, group A streptococci, anaerobic organisms (peptostreptococci, *Bacteroides*), and *Clostridium perfringes*. NF is rarely attributed to other pathogens [3]. Only a few cases of non-typhoid *Salmonella* NF have been reported to date. Herein, we report a case of NF caused by *Salmonella typhimurium* followed by a superimposed infection with *Fusarium* SSTI.

## Case presentation

### Background and presentation

**Fig. 1:**
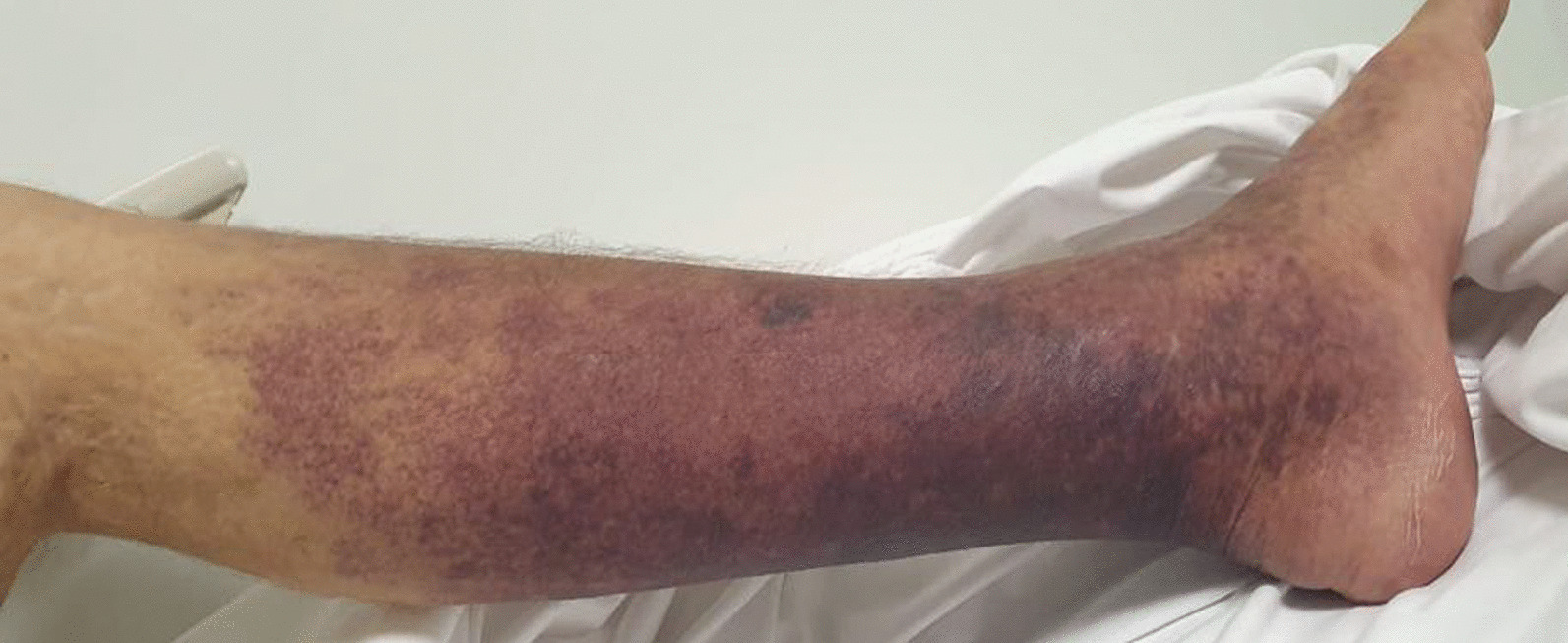
Left lower extremity violaceous discoloration at presentation

A 20-year-old Caucasian male patient presented to the emergency department (ED) with 2-day history of watery, nonbloody diarrhea, high-grade fever, and 1-day history of painful left lower extremity violaceous discoloration (Fig. 1). The patient’s past medical history was pertinent for hypoplastic right heart syndrome (HRHS) with atretic tricuspid and pulmonic valves, large atrial septal defect, and hypoplastic right ventricle status post multiple interventions, namely right modified Blalock–Taussig shunt, Glenn procedure, failed fenestrated Fontan procedure with an obligatory right to left shunt, and dual-chamber pacemaker insertion. He suffers from severe left-sided heart failure (estimated left ventricular ejection fraction of 20–24%), for which he is maintained on daily bisoprolol, enalapril, furosemide, hydrochlorothiazide, spironolactone, and warfarin with recurrent hospitalizations for heart failure exacerbation. He had been taking oral prednisone 20 mg two times daily for the past 4 months for a suspected diagnosis of protein-losing enteropathy in the setting of persistent hypoproteinemia and hypoalbuminemia. The patient is maintained on prednisone owing to the unavailability of oral budesonide in Lebanon because of the major economic crisis the country is facing and the tremendous burden on the healthcare sector and medication shortage. Other medical comorbidities included congestive hepatopathy and hypothyroidism, for which he is treated with levothyroxine 50 mcg daily. Of note, the patient is a nonsmoker, does not consume alcohol, and denied the use of any recreational drugs.

On the day of his presentation, the patient noticed a small area of violaceous discoloration on his left lower extremity (LE) shin that progressed rapidly with worsening tenderness and severe pain requiring opioids for pain relief. He denied recent trauma or penetrating injury at the site of the left LE. No animal contact was reported. He recalled consuming poultry-containing meals with mayonnaise sauce 4 days before the diarrhea onset. On review of systems, the patient noted dark-colored urine for the past 2 days and decreased appetite.

Upon presentation to the ED, the blood pressure was 70/30 mmHg, the heart rate was 95, the respiratory rate was 18, and the oxygen saturation was 81% on room air (his baseline oxygen saturation was around 82% due to right-to-left shunt and Fontan anatomy). Patient was conscious but ill-appearing with no focal neurological deficit. Examination of the left lower extremity revealed severe tenderness, extensive erythema, bluish-violaceous discoloration more accentuated on the lateral aspect of the leg reaching below the knee, weak peripheral pulses, and normal foot dorsal and plantar flexion (Fig. 1). Chest and abdominal examination was relevant for bibasilar crackles on auscultation and increased abdominal girth along with hepatomegaly; otherwise the abdomen was soft and nontender. Two sets of peripheral blood cultures were taken, and a stool sample was sent for culture. Meanwhile, the patient received an intravenous (IV) fluid bolus of 250 mL normal saline 0.9% and was started on a norepinephrine drip along with broad-spectrum antibiotics in view of his immunosuppression and shock status. IV meropenem 1 g every 8 h, clindamycin 900 mg every 8 h, and vancomycin 1 g every 8 h for possible NF were initiated. Oral azithromycin 1 g every 24 h was started for adequate salmonellosis coverage as it was suspected given exposure history. A stress dose of intravenous hydrocortisone 100 mg every 8 h was also given. Relevant laboratory work-up on presentation is summarized in Table 1.

**Table 1:** Relevant laboratory work-up on presentation. Of note, the patient’s baseline serum creatinine was 0.5–0.6 mg/dL and his last proBNP level 8 weeks prior to presentation was 5000 pg/mL

	Value	Reference range
White blood cell count	8100/cu.mm	4000–11,000/cu.mm
Neutrophils	91%	40–65%
Platelets	50 600/cu.mm	150,000–400,000/cu.mm
Serum creatinine	0.9 mg/dL	0.6–1.2 mg/dL
Serum sodium	123 mmoL/L	135–145 mmoL/L
Total bilirubin	7.5 mg/dL	0–1.2 mg/dL
Direct bilirubin	6.1 mg/dL	0–0.3 mg/dL
Serum albumin	15 g/L	36–53 g/L
Alanine transaminase	127 IU/L	0–65 IU/L
Aspartate transaminase	77 IU/L	0–50 IU/L
Lactic acid (venous)	4.49 mmoL/L	0.55–2.2 mmoL/L
Creatine phosphokinase	406 IU/L	20–205 IU/L
NT-proB-type natriuretic peptide (ProBNP)	14,931 pg/mL	≤ 63 pg/mL
C-reactive protein	277 mg/L	0–2.5 mg/L
Serum procalcitonin	86.2 ng/mL	≤ 0.05 ng/mL

### Investigations and management

NF was high on the differential diagnosis, which also included gas gangrene, necrotizing myositis, cellulitis, and toxic shock syndrome. The plastic and reconstructive surgical team was consulted, and a computed tomography (CT) angiography of the left LE was obtained. Imaging showed cutaneous and subcutaneous soft tissue thickening involving the left leg, suggestive of cellulitis and diffuse body wall edema with no radiological evidence of necrotizing fasciitis and no vascular filling defect. Lower extremities venous duplex showed no evidence of deep or superficial venous thromboembolism.

The patient was transferred to the medical intensive care unit (ICU) as he was deemed at high risk for general anesthesia given his cardiac condition. Serial follow-up examinations showed no further progression of the lower extremity discoloration compared with his initial presentation; however, the patient required the use of two vasopressors (norepinephrine and epinephrine) delivered through an urgently inserted jugular central venous catheter during the first 24 h of his hospital stay for septic and possible cardiogenic shock. Moreover, he had increased oxygen requirements reaching 10 L/min delivered through a face mask to maintain his baseline oxygen saturation. On day 1 of hospitalization, Gram-negative rods were recovered from the two sets of blood cultures taken at presentation. Broad-spectrum antibiotics regimen was maintained in the setting of ongoing septic shock and possible polymicrobial SSTI. The patient started to show hemodynamics improvement within 48 h from admission, and pressors were completely stopped on day 6 of hospitalization. The Gram-negative rods in the blood were identified as *Salmonella typhimurium*. Antibiotic susceptibility was tested by standard disk diffusion method as per Clinical Laboratory Standards Institute guidelines. The isolate was susceptible to ampicillin, ceftriaxone, cefotaxime, ceftazidime, ciprofloxacin, and trimethoprim/sulfamethoxazole. The antimicrobial regimen was modified to ceftriaxone 2 g IV every 24 h and oral azithromycin 1 g every 24 h as a dual treatment for severe *Salmonella* bacteremia with deep-seated infection [4]. Although the patient showed significant clinical and hemodynamic improvement, the lower extremity discoloration showed no improvement, and multiple serous fluid-filled bullae developed. A fluid sample was taken under sterile conditions from one of the LE bullae and was incubated in aerobic and anaerobic blood culture bottles that later grew *Salmonella typhimurium*, hence confirming the diagnosis of *Salmonella* non-typhi bacteremia with associated cellulitis. No *Salmonella* species were recovered from the stool culture taken on the day of presentation. Repeated central and peripheral blood cultures were negative at day 4 of presentation. After the vasopressors were stopped, oral azithromycin was stopped, and IV ceftriaxone was kept, treating *Salmonella* bacteremia in an immunocompromised patient with a life-threatening presentation. Transthoracic cardiac ultrasound showed no evidence of valvular vegetation. Transesophageal cardiac ultrasound was not possible, given his cardiac and respiratory status.

Direct hyperbilirubinemia and slightly elevated liver enzymes were attributed to the shock state and improved on follow-up laboratory workup. Hypervolemic hyponatremia improved with a low-dose continuous infusion of IV furosemide (5 mg/h) for 2 days. Thrombocytopenia on presentation was likely sepsis related and improved subsequently.

**Fig. 2:**
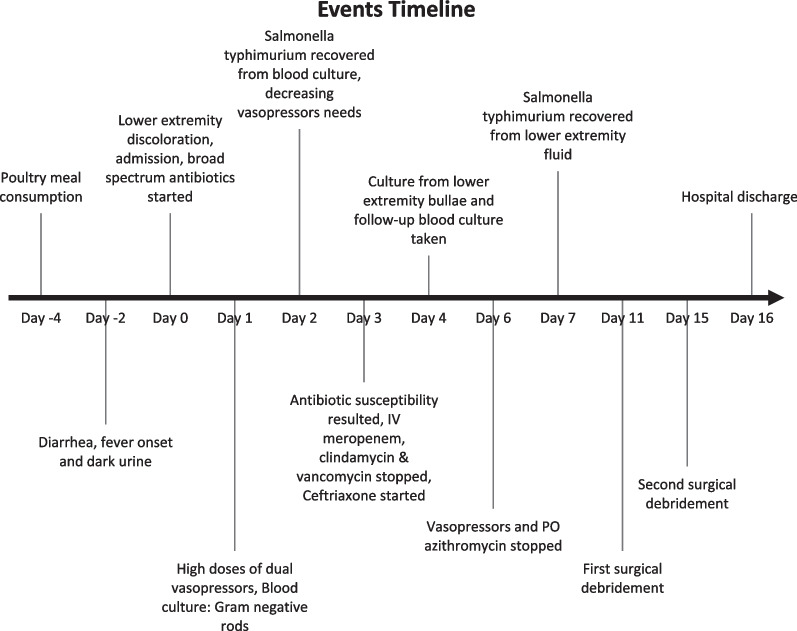
Timeline of clinical and paraclinical events of a 20-year old patient with *Salmonella typhimurium* necrotizing fasciitis

The patient was then transferred to the regular medical ward. On days 11 and 15, he underwent wide surgical debridement, excision of necrotic tissues, and thorough irrigation under regional anesthesia, as conservative management alone had failed to improve his LE condition. Intraoperative tissue cultures taken during the two surgeries showed Gram-negative rods on Gram staining and grew *Salmonella typhimurium* with similar susceptibility patterns to the one isolated in blood. Surgical pathology confirmed the diagnosis of necrotizing fasciitis. The patient was discharged on day 16, and he was prescribed oral ciprofloxacin 500 mg two times daily for an additional month to complete a total duration of 6 weeks and an oral prednisone taper regimen over 2 weeks with regular follow-up. Figure 2 summarizes the timeline of the events.

### Follow-up

Twenty-three days later, the patient underwent a third surgical debridement for necrotic tissues because of poor wound healing with the application of a dermal regeneration template. Intraoperative bacterial tissue culture showed no evidence of *Salmonella typhimurium*; however, the pathologic specimen showed invasive fungal organisms with a superficial fungal ball. Then, intraoperative tissue fungal culture grew *Fusarium* species. Blood cultures were taken subsequently and showed no evidence of bacteremia or fungemia. The patient was started on oral voriconazole 200 mg two times daily to cover superimposed *Fusarium* SSTI. The patient completed the ciprofloxacin course and was kept on oral voriconazole until undergoing skin grafting. A follow-up transthoracic cardiac ultrasound showed no evidence of endocardial involvement.

In the setting of invasive *Salmonella*, fungal SSTI, and poor wound healing, the patient was investigated for the presence of immunodeficiency. He was found to have low serum immunoglobulin G (IgG) (3.3 g/L; reference range 7–16 g/L), and blood immune profile by flow cytometry showed a decrease in T cytotoxic lymphocytes, T helper lymphocytes, natural killer cells, and B lymphocytes. He was referred to an immunology clinic for management and follow-up, and he received a single dose of 1 g/kg of intravenous immunoglobulin.

Six weeks following his last surgical debridement, the wound showed adequate healing, and the patient underwent skin grafting, after which oral voriconazole was stopped. No treatment-related side effects were identified during hospital stay and frequent clinical follow-ups.

The patient was followed up every 2 weeks in plastic surgery clinics for the first 3 months after the last surgical debridement, then monthly. Six months follow-up showed adequate wound healing.

## Discussion and conclusion

*Salmonella* species usually cause gastroenteritis, especially in developing countries, where 5% of the cases are usually complicated by secondary bacteremia, especially among immunocompromised patients [5]. Focal nontyphoidal *Salmonella* infections are relatively rare, representing around 6% of *Salmonella* infections, and may affect any organ [6, 7]. Despite its unusualness, its prevalence varies according to host factors. In fact, it is three to four times more prevalent among immunocompromised patients in comparison with the general population [6, 7]. The most common sites of focal *Salmonella* infection are bones, joints, urinary tract, intraabdominal cavity, and rarely skin and soft tissues [6]. In fact, SSTI secondary to *Salmonella* non-typhi is seen in around 1.5% of *Salmonella* non-typhi cases [8] with a wide spectrum of clinical manifestations ranging from pustular lesions to subcutaneous abscesses and very rarely NF [6, 9–11].

Necrotizing fasciitis is a serious and potentially life-threatening soft tissue infection that should be promptly handled. A multidisciplinary team should be involved in managing this aggressive condition while continuously monitoring patient hemodynamics in a controlled setting [12]. NF is a surgical emergency requiring wide and extensive debridement and reconstructive surgery [13]. Multiple surgical debridements are usually warranted until tissue necrosis ceases and the growth of viable tissue is observed. The amputation of an involved limb should also be considered in the setting of irreversible necrosis and widespread gangrene with hemodynamic compromise [14]. After the initial debridement, thorough wound follow-up is warranted with daily antibiotic dressing change, as hemodynamic instability may lead to progressive skin necrosis. The patient may return as often as necessary for further surgical debridement.

NF is usually a polymicrobial entity; hence, broad-spectrum antimicrobial agents covering aerobic Gram-positive, Gram-negative organisms and anaerobes should be started with no delay. A reasonable regimen includes a combination of penicillin G, aminoglycoside, and clindamycin, a potent suppressor of bacterial toxin synthesis. The maximum doses of the antibiotics should be used and adjusted according to the patient’s weight, kidney and liver functions [15].

In the present case report, we describe a rare incidence of monomicrobial nontraumatic *Salmonella typhimurium* NF of the lower extremity in a patient with multiple comorbid cardiac anomalies, prolonged steroid use, and immunodeficiency who presented with *Salmonella* bacteremia and improved on pathogen-directed antibiotics along with multiple surgical debridements. Only a few cases in the literature discuss complicated SSTI secondary to nontyphoidal *Salmonella* infections that commonly present in patients with predisposing immunocompromising conditions such as systemic lupus erythematosus, chronic steroids use, or immunomodulatory medications [16]. Despite the high mortality associated with NF, a case series by Khawcharoenporn et al. in 2006 found that five out of the six reported cases of *Salmonella* NF survived after a combination of prompt medical and surgical management, such as our case (16). Moreover, the presented case was complicated by a superimposed fungal infection with *Fusarium* species that was successfully managed with surgical debridement and oral voriconazole. Hence, a nonhealing wound in a similar setting should prompt consideration of superimposed opportunistic infections such as fungus.

Despite its novelty in describing a rare entity with an unorthodox management plan dictated by the multiple comorbid conditions in our patient and his clinical evolution, this manuscript remains a case report with limited possibility of validity generalization. However, it may serve as a potential management plan in similar rare cases in light of the scarcity of evidence. Further larger-scale observational studies are required to establish a consensus on the management of this rare entity in frail patients.

NF is a serious infection of soft tissues associated with high morbidity and mortality rates if not managed in a timely manner. The initial management should include a broad-spectrum antibiotic regimen, which would be ultimately adjusted according to culture results. Even though NF is most commonly related to polymicrobial infection, it is important to maintain a high index of suspicion for rare causative entities such as *Salmonella* non-typhi SSTI, in particular in immunosuppressed patients in whom a delay in diagnosis and management may cause significant morbidity and mortality.

## Data Availability

All data pertaining to this patient are included in this report.

## Abbreviations

NF: Necrotizing fasciitis
SSTI: Skin and soft tissue infections
LE: Lower extremity
ED: Emergency department
HRHS: Hypoplastic right heart syndrome
IV: Intravenous
CT: Computed tomography
ICU: Intensive care unit
Mg/h: Milligram per hour
IgG: Immunoglobulin G
g/L: Gram per liter
g/kg: Gram per kilogram
ProBNP: NT-proB-type natriuretic peptide
